# Emotional violence exerted by intimate partners and postnatal depressive symptoms among women in Vietnam: A prospective cohort study

**DOI:** 10.1371/journal.pone.0207108

**Published:** 2018-11-09

**Authors:** Nhi Tho Tran, Hanh Thi Thuy Nguyen, Hinh Duc Nguyen, Toan Van Ngo, Tine Gammeltoft, Vibeke Rasch, Dan W. Meyrowitsch

**Affiliations:** 1 Institute of Preventive Medicine and Public Health, Hanoi Medical University, Khuong Thuong, Dong Da, Hanoi, Vietnam; 2 Department of Anthropology, University of Copenhagen, Copenhagen K, Denmark; 3 Department of Obstetrics and Gynecology, Odense University Hospital, Odense C, Denmark; 4 Dept of Clinical Research, University of Southern Denmark, Odense M, Denmark; 5 Department of Public Health, Faculty of Health Sciences, University of Copenhagen, Copenhagen, Denmark; Hogskolen i Oslo og Akershus, NORWAY

## Abstract

**Background:**

Previous studies have shown a relation between intimate partner violence (IPV) and postpartum depression (PPD). However, these studies have primarily focused on physical and sexual violence as predictors for postpartum depression and little attention has been given to emotional violence (EV), despite emotional violence having been well reported as the most common type of violence experienced by women. This present study aimed to investigate the association between various types of emotional experience during life with present partner and postnatal depressive symptoms among women in Vietnam.

**Methods:**

A total of 1,274 pregnant women were recruited from 24 communities in the Dong Anh District, Hanoi, Vietnam. They were interviewed four times: (a) at enrolment (before week 24 of pregnancy); (b) at a gestational age of 30–34 weeks; (c) 24–48 hours after delivery; and (d) 4–12 weeks after delivery. Emotional violence and postnatal depressive symptoms were measured using a questionnaire developed by the World Health Organization (WHO) and the Edinburgh Postpartum Depression Scale (EPDS), respectively.

**Results:**

A total of 639 (50.4%) women experienced at least one type of emotional violence with their present partner, whereas 104 women (8.2%) experienced postnatal depressive symptoms. Women exposed to emotional violence were more likely to experience postnatal depressive symptoms (OR = 3.15; 95%CI: 1.17–8.51). Other statistically significant predictors of increased postnatal depressive symptoms included type of employment, lack of family support after delivery, lower level of education, husband’s preference for a specific sex of child, presence of mental disorder, and depression during pregnancy.

**Conclusions:**

Among Vietnamese women, there was a statistically significant association between exposure to emotional violence with their present partner and postpartum depression. The findings indicate an urgent need for screening for all acts of emotional violence as risk factors for postnatal depressive symptoms.

## Introduction

Violence exerted against women by their intimate partner is recognized as an important public health problem worldwide [1,2]. According to WHO, Intimate Partner Violence includes physical, sexual, and emotional violence as well as controlling behaviours by current partners or ex-partners [3]. Emotional violence is the most common type of IPV [4]. The results of a multi-country study conducted by WHO (2010) on women's health and domestic violence against women showed that within the past 12 months, between 20% and 75% of women had experienced one or more of types of EV exerted by their intimate partner [3], and 30% of women experienced, physical and/or sexual intimate partner violence during their lifetime [5]. In Vietnam, research has documented frequent occurrences of EV; results of a nationwide study showed that the 12-month and lifetime prevalence of exposure to EV were 25% and 54%, respectively. The 12-month and lifetime prevalence for exposure to physical violence were 6% and 32%, whereas the prevalence for exposure to sexual violence were 4% and 10%, respectively [6].

Globally, EV and other forms of IPV pose serious threats to women’s health, dignity and autonomy, while also affecting children, families and communities [3,7,8]. Emotional violence is also associated with detrimental consequences for women's mental health [4] and plays a precipitating role in women’s depression [9]. Postpartum depression (PPD) affects 10–15% of mothers within the first year after giving birth [10]. Depressive women are more likely to display negative feelings such as sadness, anxiety, tension and anger [11], and suicidal ideation [12]. The direct evidence suggests that screening pregnant and postpartum women for depression may reduce depressive symptoms in women [13]. However, in Vietnam, maternity services have traditionally managed the physiological processes of childbirth and are separated from mental health services with little interaction between the two, except in the referral of complex cases. Tertiary inpatient psychiatric units are available for cases of severe mental illness and a national program provides free medications for sufferers of psychosis and epilepsy [14]. Several risk factors have been associated with PPD, including genetic predisposition, low education level, low income, unemployment, lack of social support, stress and intimate partner violence [15]. There is a wealth of research on PPD and its associated risk factors, but the majority of studies use cross-sectional surveys. Only a few studies have been based on a cohort study design [15]. In Vietnam, there are a limited number of studies focusing on this topic and they have primarily been conducted with small sample sizes in Ho Chi Minh and Hue [16,17]. Previous research conducted in Hanoi focused on social, cultural, and religious factors as well as the relationship between being physically abused and depression [18,19].

Some studies have assessed the association between exposure to partner violence and risk of postnatal depressive symptoms during pregnancy [20,21]. However, these studies have primarily focused on physical and sexual violence; and, little attention has been given to women being exposed to emotional violence [9]. Moreover, it has previously been recommended that future research should focus on how emotional violence affects women’s mental health after delivery [22,23]. According to a study conducted in Vietnam, emotional violence exerted by the intimate partner was overlooked, because it was regarded as an acceptable way to treat women and therefore rarely perceived as a general problem [18]. The findings affirmed the need to conduct further research into the effects of emotional violence on the mental health of women after delivery. Moreover, a detailed understanding of what constitutes emotional violence, and strategies for reducing this violence during pregnancy and after giving birth, is important for improving mental health and preventing negative consequences for the health of women and their children.

The aim of the present study was to measure the association between different types of emotional violence exerted by a woman’s current intimate partner and postnatal depressive symptoms among women in Northern Vietnam.

## Materials and methods

### Study design, study setting and sampling

This study is a part of the larger interdisciplinary research project The Impact of Violence on Reproductive Health in Tanzania and Vietnam (PAVE) that explores the impact of partner violence on women’s reproductive health. The PAVE project study design included a quantitative and a qualitative component. A total of 1,337 pregnant women were invited at baseline to participate in the study, and 1,274 women completed all interviews and were included in the final sample. Women were recruited in their early pregnancy from all communities in Dong Anh District from May 1, 2014 to August 30, 2015.

Dong Anh District is located 20 kilometres east of Hanoi city and covers an area of 182.3 km2. The district has a total population of approximately 390,000 people. The district is divided into 23 rural communities and community-level town. In Vietnam, there are three kinds of third-level (community-level) administrative subdivisions: the rural community (Vietnamese: xã), the community-level town (Vietnamese: thị trấn), and the ward (Vietnamese: phường, literal meaning: urban subdistrict). In Vietnam a rural community is referred to as a xã and urban community is referred to as Thị trấn, urban townships. However many communities, particularly large urban ones with provincial status, will be divided into wards which are known as phường”. The population is engaged in agricultural production, livestock breeding (mainly in the rural areas) and in local industrial production. There are two major hospitals in the district (Dong Anh Hospital and Bac Thang Long Hospital). These two hospitals facilitate the majority of the antenatal care services and deliveries in the district. In these two hospitals combined, an estimated total of 11,600 women annually seek antenatal care and delivery.

### Data collection

Each woman was assigned a case manager and interviewed four times: at enrolment (at a gestational age of less than 24 weeks); at a gestational age of 30–34 weeks; 24–48 hours after delivery; and 4–12 weeks after delivery. Inclusion criteria for participants included: a) a permanent address in the Dong Anh District; and b) presenting with a gestational age of less than 24 weeks at the time of enrollment. Exclusion criteria for participants included: a) lack of consent regarding participation; and b) women with severe mental illness.

Prior to the recruitment of participants, the local Population Center in collaboration with local health authorities informed all pregnant women in the district about the research project. This information included an explanation of the purpose of the study and details regarding access to free ultrasound scanning. The pregnant women were examined by ultrasound scanning performed by a Medical Doctor (research team member) in order to obtain an accurate measure of gestational age. This examination, which provided information regarding the gestational age and the health of the fetus, was perceived as a benefit and therefore functioned as an incentive for the pregnant women to participate in the research project. In addition, women received a total of 40,000 Vietnamese Dong for completion of the first and second interview. In the third interview, they received diapers for newborns and for the final interview, they received a large bath towel for their baby. Six interviewers were recruited among the staff of the Population Center in the Dong Anh District and they received training in interviewing skills. They received comprehensive training on the concepts of gender, gender discrimination, inequality, domestic violence and how to assist and support participants who were exposed to violence.

On a monthly basis, the Population Center received a list of all pregnant women with an estimated gestational age of less than 22 weeks. The women were all registered in the Dong Anh District and included details regarding their name, place of birth, and residential address. These lists were used in the present study as a sampling frame for the recruitment of pregnant women. The pregnant women were invited to two major hospitals or to one of the 24 Community Health Stations (CHS) located in the Dong Anh District. Informed consent was obtained from all women. Hereafter, the gestational age was determined through the use of ultrasound scanning performed by an obstetrician. The women who fulfilled the inclusion criteria were invited to participate in the first interview, which took place in a separate room specifically allocated for the research project. During this interview, the interviewer obtained general information about the women, including their age at first pregnancy and their partner’s preference regarding the sex of the child. Subsequently, the women were given a project registration card and the time and place of the second interview. The second interview was held either at the women’s home or at a CHS depending on the women’s preference. During this interview, we obtained information regarding the exposure to different types of emotional, physical and sexual violence experienced during their time with their present partner. The third interview was held in the hospital where the delivery took place and it was conducted in a private hospital room. Here the interviewers collected information regarding the actual gestational age at birth. During the third interview, a new appointment was made regarding the time and place of the fourth interview. This fourth interview was held at the home of the women or, if needed for confidentiality reasons, in another place chosen by the women. During this interview, signs of depression were assessed and information regarding family support after delivery was collected.

### Measurements

Postnatal depressive symptoms were assessed by the use of the Edinburg Postpartum Depression Scale (EPDS) [24]. The EPDS is based on a 10-item questionnaire with four response categories scored from 0 to 3. The categories yield a range of 0–30, whereby the greatest values indicate pronounced signs of depression. The EPDS has been translated into Vietnamese and used, and validated in a previous study conducted in Vietnam [25]. The EPDS scale is regarded as a valid tool for screening for depression [26]. The specific cut-off value of 10, applied in the present study, has been validated and the results indicate that the Vietnamese version of the EPDS using this specific cut-off point, is a reliable and valid screening tool for perinatal depression [27,28]. Hence, in the present study, mothers who had an EPDS total score of 10 or higher were defined as presenting with postnatal depressive symptoms.

For the measurement of IPV we used a slightly modified version of the Multi-Country Study on Women’s Health and Life Experiences Questionnaire developed by the WHO with a specific focus on IPV [29]. In brief, emotional violence was assessed as being insulted or made to feel bad about oneself; belittled or humiliated in front of other people; scared or intimidated on purpose; and having received threats intended to hurt a person one cared about. The questions addressed the entire period spent with the present partner. In the present study, exposure to emotional violence was defined as being exposed to at least one out of the four above-mentioned types of emotional violence. Physical violence was assessed as being slapped, being hit with a fist or some other object that could hurt the participant; being pushed, shoved or pulled by the hair; being dragged, beaten, choked or burned purposely; or being threatened with a weapon such as a gun, a knife or any other weapon. Sexual violence was assessed as having sexual intercourse against the participant's will, using physical force for sexual intercourse, or forcing the participant to engage in sexually degrading acts. Exposure to physical and sexual violence was defined as being exposed to at least one type of physical and sexual violence, respectively.

### Statistical analysis

The data was double entered in EPI-DATA 3.1 for quality control and data analyses were performed in STATA version 12 for Windows.

Bivariate and multivariate analyses were used to measure the strength of the association between specific types of emotional violence and signs of PPD. The multivariate analyses included adjustment for physical and sexual violence, age of woman, level of education, occupational status of woman, partner’s preference regarding the sex of the child, gestational age at birth, age of woman at first pregnancy and family support after delivery.

For the multivariate analyses, the determinants were selected at each level according to their statistical significance (value p<0.2) to avoid excluding a potential confounding factor [30] and based on empirical literature. A p-value <0.05 was considered statistically significant. The results were presented as odds ratios (ORs) and 95% confidence intervals (95%CI) around the respective ORs.

The Population Attributable Risk (PAR) was calculated in order to measure the proportion of postpartum depression in the population that is due to the exposure to emotional violence.

### Ethical considerations

We followed WHO’s ethical and safety recommendations for researching domestic violence against women [31]. An informed written consent was obtained from all women prior to the first interview. In this study, two participants were aged 17 and accompanied by their mother, who also signed the consent form. The participants were interviewed in private rooms or, if preferred by participants, in an alternative place that they selected. The confidentiality of participants was protected at all times. During proposal development and as part of the project work, meetings with relevant health providers, legal authorities, the police, women's organizations and religious organizations were conducted. All women who reported exposure to IPV were provided with a list of organizations supporting victims of violence and the participants informed us that they could keep the list in a safe place. An ethical approval for the research was obtained from the Research Ethics Committee at the Hanoi Medical University (permit no 137, November 29, 2013).

## Results

The original sample comprised a total of 1,350 pregnant women, of whom 1,337 met the inclusion criteria and consented to participation in the study. The response rate was 95.4%. A total number of 63 women were subsequently lost to follow-up a majority of these participants had completed higher levels of education via college or university, accounting for 46% of all participants. Approximately one-third of the participants were employed during the study period (30%). Regarding reproductive history of the participants, 12.7% of the women had a history of miscarriage, 6.3% had a history of stillbirth and 6.3% had a history of abortion. The most common type of violence reported was emotional violence (49.2%). Nearly 8% of the women had experienced physical violence and 9.5% reported sexual violence with present partner. Emotional violence was experienced by 25.4% and 3.2% reported physical violence during their pregnancy. Finally, 11.1% women presented with an EPDS score of 10 and above).

A total number of 1,274 participants were included in the final analysis. Descriptive characteristics of the study participants are shown in **Table 1**. Most participants were aged 25 years and above (65%). More than half of the participants (52.1%) were born in a different community/district/province/city as compared to their present residential location. Almost one-third of the participants were government/private company employees (32%), almost half were university or college graduates (43.7%) and nearly all (99.5%) were married and living with their partner. The majority of the women were living with their family in-law (67.2%).

**Table 1:** Descriptive characteristics of study participants.

Characteristics	No. of women	% of total
**Age** (years) **(n = 1,274)**		
<24	446	35.0
25–29	468	36.7
≥ 35	360	28.3
**Place of birth (n = 1,272)**		
Born in commune and still resides in the same commune of Dong Anh District	610	47.9
Born in one commune in Dong Anh District and presently live in another commune of Dong Anh District	350	27.5
Born in another district/province/city and now live in Dong Anh District	312	24.6
**Occupation (n = 1,273)**		
Government/Private company /Organization employee	408	32.0
Work in private company	349	27.4
Farmer	166	13.0
Small trade	181	14.2
Unemployed/student/*Housewife*	169	13.4
**Level of education (n = 1,274)**		
Primary school (up to grade 5 years)	24	1.9
Secondary school (grade 6–9 years)	228	17.9
High school (grade 10–12 years)	465	36.5
University/college ((>12 years)	557	43.7
**Living arrangement in relation to partner (n = 1,273)**		
Married and living together	1,267	99.5
Married but living apart	3	0.2
Living with a man, not married	2	0.2
Having a regular partner (sexual relationship), living apart	1	0.1
Living arrangement in relation to family of birth* **and in-laws (n = 1,274)**		
Living without family of birth/in-laws	356	27.9
Living with family of birth	62	4.9
Living with family in-law	856	67.2
**Combined exposure to types of emotional violence**		
Not exposed	630	49.6
One type of emotional violence	571	45.0
Two types of emotional violence	42	3.3
Three or more types of emotional violence	26	2.1
**The prevalence of postpartum depression**	**104**	**8.2%**

* Living with one and/or two biological parents

### Type of emotional violence experienced during pregnancy and life with partner

Overall, 45% women reported experiencing at least one act of emotional violence by their present partner, and 5.4% had experienced three or more acts of EV (**Table 1**). Being “scared or intimidated” was the most commonly reported act of EV reported (44.1% during life with present partner, 29% during pregnancy), followed by being “insulted or made to feel bad about themselves” (9.1% during life with present partner, 5.1% during pregnancy), and “belittled or humiliated in front of others” (2.6% during life with present partner, 1.6% during pregnancy), and “threatened to hurt woman or someone the woman cared about” (2.5% during life with present partner, 1.3% during pregnancy).

### Frequency of acts of emotional violence

Among various acts of EV experienced by women during their pregnancy and lifetime with their partners, being scared or intimidated on purpose was the most common (29% and 44.1%, respectively). Of those, the majority had experienced such acts of EV between 2–5 times (**Table 2**).

**Table 2:** Acts of emotional violence experienced during pregnancy and life with partner.

Type of emotional violence	During pregnancy				During life with partner			
	Violence exp. n (%)	Number of events			Violence exp. n (%)	Number of events		
		Once n (%)	2–5 times n (%)	>5 times n (%)		Once n (%)	2–5 times n (%)	>5 times n (%)
Insulted woman or made woman feel bad about themselves	65 (5.1)	11 (16.9)	46 (70.8)	7 (12.3)	116 (9.1)	30 (25.9)	72 (62.1)	14 (12.0)
Belittled or humiliated woman in front of other people	20 (1.6)	4 (20.0)	11 (55.0)	5 (25.0)	33 (2.6)	9 (27.3)	15 (45.4)	9 (27.3)
Done things to scare or intimidate woman on purpose	370 (29.0)	61 (16.5)	260 (70.3)	49 (13.2)	560 (44.1)	103 (18.4)	313 (55.9)	144 (25.7)
Threatened to hurt woman or someone the woman cared about	16 (1.3)	6 (37.5)	9 (56.2)	1 (6.3)	32 (2.5)	12 (37.5)	14 (43.7)	6 (18.8)

### The prevalence distribution of emotional violence during pregnancy and during life with present partner

Overall, women reported that exposure to at least one act of emotional violence exerted by an intimate partner was common (50.4% during life with present partner; 32.3% from 30 to 34 weeks gestation; 24.7% before 24 weeks gestation, and 43.8% pre-pregnancy (**Table 3**)).

**Table 3:** The prevalence distribution of various types of intimate partner violence experienced during pregnancy and during life with present partner.

Type of partner violence	No. of women	% of total (95%CI)
**Pre-pregnancy**		
Emotional violence	558	43.8 (41.1–46.5)
Physical violence	148	11.6 (9.9–13.4)
Sexual violence	108	8.5 (6.9–10.0)
**Before 24 weeks gestation**		
Emotional violence	314	24.7 (22.3–27.0)
Physical violence	40	3.1 (2.2–4.1)
Sexual violence	63	4.9 (3.8–6.1)
**30–34 weeks gestation**		
Emotional violence	411	32.3 (29.7–34.8)
Physical violence	45	3.5 (2.5–4.5)
Sexual violence	125	9.8 (8.2–11.4)
**During life with present partner**		
Emotional violence	639	50.2 (47.4–52.9)
Physical violence	163	12.8 (11.0–14.7)
Sexual violence	161	12.7 (10.9–14.5)

### The prevalence of postnatal depressive symptoms

A total of 104 women (8.2%) presented with an EPDS score of 10 and above and were defined as having signs of PPD **(Table 1).**

### The association between exposure to emotional violence during life with their present partner and postnatal depressive symptoms

The results of the crude analyses (**Table 4**) showed that exposure to a combination of three or more types of emotional violence were the strongest significant predictor for postnatal depressive symptoms (OR = 8.89; 95%CI: 3.58–22.08). This was followed by a combination of two types of emotional violence (OR = 6.25; 95%CI: 2.81–13.89), and finally one type of emotional violence (OR = 2.17; 95%CI: 1.37–3.44).

**Table 4:** Associations between risk factors and postnatal depressive symptoms.

Factor	No. of women in each group (% of women with signs of depression)	Bivariate analysis		Multivariate analysis	
		OR (95%CI)	p-value	AOR (95%CI)	p-value
**Combined exposure to types of emotional violence**					
Not exposed	630 (4.8)	1		1	
One type of emotional violence	571 (9.8)	2.17 (1.37–3.44)	0.001	2.28 (1.35–3.86)	0.002
Two type of emotional violence	42 (23.8)	6.25 (2.81–13.89)	0.000	3.15 (1.17–8.51)	0.023
Three or more types of emotional violence and above	26 (30.8)	8.89 (3.58–22.08)	0.000	3.16 (0.83–12.03)	0.091
**Physical violence**					
Yes	163 (12.9)	1.82 (1.09–3.04)	0.021	0.64 (0.30–1.35)	0.243
No	1,106 (7.5)	1		1	
**Sexual violence**					
Yes	161 (11.8)	1.61 (0.95–2.73)	0.077	1.11 (0.59–2.07)	0.754
No	1,108 (7.7)	1		1	
**Age of women** (years)					
<25	446 (8.7)	1.12 (0.74–1.70)	0.578	-	**-**
≥25	828 (7.9)	1			
**Occupation of women**					
Small trade	181 (4.9)	1		1	
Employed by government, private company or organization	408 (10.5)	2.25 (1.07–4.72)	0.032	3.73 (1.55–8.96)	0.003
Farmer	166 (13.9)	3.07 (1.38–6.85)	0.006	2.79 (1.14–6.80)	0.024
Worker	349 (5.7)	1.16 (0.52–2.61)	0.716	1.12 (0.45–2.74)	0.806
Unemployed/student	169 (5.3)	1.08 (0.42–2.78)	0.881	1.36 (0.46–3.95)	0.570
**Level of education**^**1**^					
University/college	557 (6.5)	1		1	
High school	465 (8.4)	1.32 (0.83–2.12)	0.241	2.17 (1.21–3.89)	0.009
Primary school/Secondary school	252 (11.5)	1.88 (1.13–3.15)	0.016	3.55 (1.74–7.25)	0.000
**Husband’s preference for a specific sex of child**^**2**^					
No preference	423 (5.4)	1		1	
Preference for son	575 (9.7)	1.88 (1.14–3.10)	0.014	1.78 (1.01–3.13)	0.046
Preference for girl	270 (9.3)	1.77 (0.99–3.19)	0.056	1.74 (0.89–3.39)	0.099
**Gestational age at delivery (weeks)**					
≥37	1,200 (7.7)	1		1	
<37	57 (17.5)	2.56 (1.25–5.24)	0.010	2.20 (0.96–5.03)	0.060
**Age of women at first pregnancy**					
≤20	258 (5.8)	1		1	
>20	1,015 (8.8)	1.55 (0.89–2.74)	0.125	2.88 (1.44–5.77)	0.003
**Family support after delivery**^**3**^					
Yes	1,028 (5.7)	1		1	
No	245 (18.4)	3.69 (2.44–5.61)	0.000	3.53 (2.19–5.70)	0.000
**Age of women** (years)					
<24 years	446 (8.7)	1.23 (0.73–2.06)	0.79	2.15 (1.14–4.05)	0.018
25–29 years	468 (8.3)	1.17 (0.69–1.96)	0.059	1.46 (0.79–2.68)	0.217
>30 years	360 (7.2)	1		1	
**Pregnancy complications**					
Yes	122 (10.7)	1.37 (0.74–2.53)	0.317	1.18 (0.58–2.39)	0.644
No	1,135 (8.0)	1		1	
**Mental health status**					
No	1,028 (6.1)	1		1	
Mental disorder	246 (16.7)	3.06 (2.01–4.67)	0.000	2.44 (1.51–3.94)	0.000
**Depression during pregnancy**					
No	1,206 (6.7)	1		1	
Yes	63 (33.3)	6.77 (3.83–11.9)	0.000	4.12 (2.06–8.21)	0.000

^1^ Level of education was grouped into primary school (up to grade 5) and secondary school (grade 6–9 years) and high school (grade 10–12) and higher education (> grade 12).

^2^Husband’s expressed preference of the sex of the unborn child (present pregnancy)

^3^Presence of at least one member of the family who has offered to take care of and support the mother and child

After adjustments for relevant factors in the full model (**Table 4**), including exposure to physical violence and sexual violence, the strongest predictor for postnatal depressive symptoms remained an exposure to a combination of two types of emotional violence (OR = 3.15; 95%CI: 1.17–8.51) and one type of emotional violence (OR = 2.28; 95%CI: 1.35–3.86).

### Other predictors for postnatal depressive symptoms

Results of the full model (**Table 4**) showed that other statistically significant predictors for postnatal depressive symptoms included being a government employee or employed by a private company; or a farmer (OR = 3.73; 95%CI: 1.55–8.96) and OR = 2.79; 95%CI: 1.14–6.80, respectively); lack of family support after delivery (OR = 3.53; 95%CI: 2.19–5.70); primary/secondary and high school education of woman (OR = 2.17; 95%CI: 1.21–3.89 and OR = 3.55; 95%CI: 1.74–7.25, respectively); pre-term birth (OR = 2.20; 95%CI: 0.96–5.03); older age at first pregnancy (OR = 2.88; 95%CI: 1.44–5.77); husband’s preference for a specific sex of child (OR = 1.78; 95%CI: 1.01–3.13); age of women <24 years old (OR = 2.15; 95%CI: 1.14–4.05), presence of a mental disorder (OR = 2.44; 95%CI: 1.51–3.94); and depression during pregnancy (OR = 4.12; 95%CI: 2.06–8.21).

Calculation of PARs in relation to depression after delivery indicated that the proportion of depression that could be attributed to emotional violence was 41.9%. Assuming that emotional violence was eliminated in the study population, this would correspond to a reduction of new cases of depression after delivery of 3.4 cases per 100 women.

## Discussion

Emotional violence experienced during life with the present partner was a strong statistically significant predictor factor for postnatal depressive symptoms. The results from the present study are in accordance with previous research carried out in Canada [9]. In the latter study, Denis *et al*. showed that women who were humiliated by their partner had 2.5 times increased risk of PPD [32]. If it is assumed that there is a causal association between emotional violence and postnatal depressive symptoms, the biological mechanism is not well understood. However, Schneiderman *et al*. suggested that emotional violence will lead to psychological pain, which again will result in increased levels of perceived stress [33]. Stress is known to trigger a cascade of changes in the body, including symptoms of fatigue, malaise and loss of appetite. These symptoms also accompany depression. Although individuals experience a variety of reactions to stress, chronic stress is known to be associated with postnatal depressive symptoms [33]. Other studies report the possibility of changes in risk behavior that may result from violence (such as alcohol intake and smoking), which also aggravate the risk of PPD [34]. Another explanation for the findings may be that exposure to emotional violence coexists in settings with a high level of dysfunctional family relations, including lack of partner support, which also may result in responses such as psychological problems [35]. This could be explained by the dynamics of maternity care in the cultural context of Vietnam. Here, women are usually supported partially or fully during the first month after delivery by family members including the mother, mother-in-law, other relatives and husband [36]. The help provided ranges from childcare to cooking, and other work, which could serve as a protective factor against depression after delivery [37].

In northern Vietnam, women who get married will most often leave their natal families and move to live with or nearby their husband’s family (marriages are patrilocal) [35]. The majority of women in our study had moved into the house of their husband’s family after getting married. It seems to be apparent that all women expect to get married with a reliable partner. This is especially important in a cultural context where kinship is patrilocal and women may end up living far from their own family and loved ones after getting married. Thus, intimate partner violence can worsen a woman’s sense of alienation and loneliness, causing her to lose courage. In this state of helplessness, women can be prone to depression [38]. Further, in cases where conflicts occur between the woman and her mother-in-law–which are ubiquitous in many parts of Asia [39]–the man will often feel culturally obliged to support his mother, a tendency that may place further strain on the marital relationship [39].

Previous studies showed that these living conditions–having to leave their own natal family behind and move into a household of strangers–set very particular conditions for the women’s marriages and their experiences of marital strain because the women’s vulnerability to emotional partner violence seemed to deepen due to the social isolation that many of them experienced [37–39].

The most common types of emotional violence exerted by the intimate partner in the present study included words or actions that scared or intimidated the woman on purpose, followed by insulting the woman or making the woman feel bad about herself. This is in accordance with previous studies reporting insult and humiliation as the most common types of emotional violence [38–40]. Hence, from a public health perspective this emphasizes the importance of increasing the focus on emotional violence due to its high prevalence and the associated psychological distress, which may result in adverse health outcomes, including mental distress and negative pregnancy related health outcomes [41]. Some acts were not perceived by the women to be violent because they were viewed as part of relationships and as manifestations of a “hot temper” and thus tolerated—for example, damaging household items. Similar behaviors could be defined differently, depending on the context and level of acceptance of the women. Some women spoke about these behaviors in terms of “talking back and forth” and “not yet violence” [6]. In such circumstances, women are not seeking help since they feel that there is no need for it or that it would not help them in their present situation. In settings where individuals and communities tolerate acts of emotional violence, it is critical to intensify efforts to better understand the consequences of such acts and to educate the population about the related adverse health outcomes.

This study indicated that the prevalence of postpartum depression in Dong Anh district was 8.2%. This prevalence is similar to the prevalence of depression disorders in Europe (8.6%) [42]. However, two recent studies carried out in Vietnam reported higher rates of 13.4% and 18.1%, respectively [17,18]. The variation in the reported prevalence of postpartum depression may be due to several factors, including the selection of the EPDS cut-off point, the sample size and the location of recruiting participants [15]. In Vietnam, the studies used different cut-off scores of EPDS ranging from 4 to 13 in order to determine the prevalence of PPD. Furthermore, time frames for measuring PPD varied considerably ranging from 4 weeks to 12 months, leading to difficulties in comparing research findings across studies. For example, the study conducted by Tran Tuan and his colleagues in Hanam Province, measured PPD at 8 weeks postpartum and used a 3/4 cut-off point on the EPDS. Fisher et al. used a 12/13 cut-off point in a study measuring PPD among women from one to six months postpartum in Hue Province.

Our study showed that a reduction of 3.4 new cases of PPD per 100 population is expected if women did not experience emotional violence (PAR = 3.4 per 100). This represents a 41.9% reduction of the incidence in the population (PAR% = 41.9%) and clearly demonstrates the crucial importance of recognizing emotional violence as a pronounced contributor to the overall burden of PPD. This calls for more investment in effective primary and secondary preventive measures with the aim of mitigating the adverse health effects of emotional violence.

Several limitations of this study should be noted. Although we have examined a number of factors that potentially influence the association between the type of emotional violence and PPD, not all relevant factors may have been included in the analysis, which may have led to residual confounding. Secondly, measurements of violence only addressed the period spent with the present partner. Since the period spent with the respective partners varied from one month to 23 years it is likely that this variation may have influenced the intensity of exposure to violence, and thereby affected the association between emotional violence and postpartum depression. Thirdly, violence and depression are sensitive topics that would likely make some women hesitant to disclose their experiences and thus lead to an under estimation of the prevalence and strength of the association between the type of emotional violence and PPD. Finally, the assessment of the outcome of interest, signs of depression, relied on self-reporting rather than clinical assessment and this may have led to an under or overestimation of postnatal depressive symptoms after delivery.

## Conclusion

Evidences from this study showed that emotional violence is common among Vietnamese women and is associated with increased risk of experiencing signs of PPD. These findings may encourage health professionals to screen for violence as a proxy factor for development of PPD, and devise appropriate and effective interventions to mitigate both exposure to emotional violence and signs of PPD. In an effort to reduce the burden of depression and other IPV-related health consequences, continued research is recommended for evaluating various forms of IPV preventive strategies.
